# Editorial board

**DOI:** 10.1080/0886022X.2020.1858558

**Published:** 2021-03-08

**Editor-in-Chief**

**Tibor Fülöp,** MD, PhD; Medical University of South Carolina, Charleston, South Carolina, USA

**Deputy Editor**

**Mihály Tapolyai,** MD, PhD; FMC Hungary, Hatvan, Hungary

**Associate Editors**

**Basic Sciences**

**István Arany,** PhD, CSc; University of Mississippi Medical Center (ret.), Jackson, Mississippi, USA

**Clinical Sciences**

**Sohail Abdul Salim,** MD; University of Mississippi Medical Center, Jackson, Mississippi, USA

**Lajos Zsom,** MD; FMC Hungary, Cegléd, Hungary

**Biostatistics and Clinical Epidemiology**

**Wisit Cheungpasitporn,** MD; University of Mississippi Medical Center, Jackson, Mississippi, USA

**Editorial Board Members:**

**Anand Achanti,** MD; Medical University of South Carolina, Charleston, South Carolina, USA

**Aysen Akalin,** MD; Eskisehir Osmangazi University, Eskisehir, Turkey

**Andrea Angioi,** MD; Azienda Ospedaliera G. Brotzu, Cagliari, Italy

**Sebastjan Bevc,** MD; University of Maribor, Maribor, Slovenia

**Ana de Lurdes Agostinho Cabrita,** MD; Faro, Portugal

**Jorge Castaneda,** MD; University of Mississippi Medical Center, Jackson, Mississippi, USA

**Orsolya Cseprekal,** MD, PhD; Semmelweis University, Budapest, Hungary

**Ersin Fadıllıoğlu,** MD; Ankara, Turkey

**Abduzhappar Gaipov,** MD, PhD; Nazarbayev University School of Medicine, Nur-Sultan City, Kazakhstan

**Mehul P. Dixit,** MD; University of Mississippi Medical Center, Jackson, Mississippi, USA

**Neville R. Dossabhoy,** MD; Louisiana State University, Shreveport, LA, USA

**Christian A. Koch,** MD, PhD; Fox-Chase Cancer Institute, Temple University, Philadelphia, PA, USA

**S. Mehrdad Hamrahian,** MD; Thomas Jefferson University, Philadelphia, Pennsylvania, USA

**Csaba Kopitko,** MD, PhD; Uzsoki Utcai Kórház, Budapest, Hungary

**Nicolas Hanset,** MD; University Hospital Saint-Luc, Brussels, Belgium

**Jolanta Malyszko,** MD, PhD, Medical University of Warsaw, Warsaw, Poland

**Miklos Z. Molnar,** MD, PhD; University of Tennessee Health Science Center, Memphis, TN, USA

**Lovelesh Nigam,** MD; Dr. H.L. Trivedi Institute of Transplantation Sciences, Asarwa, Ahmedabad, India

**Ken Sakai,** MD, PhD; Toho University Faculty Medicine, Tokyo, Japan

**Karim M. Soliman,** MD, MSc; Cairo University, Cairo, Egypt

**Blaithin A. McMahon,** MD, PhD; Medical University of South Carolina, Charleston, South Carolina, USA

**Daniela Ponce,** MD, University of Sao Paulo State, UNESP – Botucatu, Brazil

**Vinaya Rao,** MD; Medical University of South Carolina, Charleston, South Carolina, USA

**László Rosiváll,** MD, PhD, DSc Med, Medhabil.; Semmelweis University, Budapest, Hungary

**Michael E. Ullian,** MD; Medical University of South Carolina, Charleston, South Carolina, USA

**Wisit Kaewput,** MD; Phramongkutklao Hospital, Bangkok, Thailand

**Statistical Consultant Advisor:**

**Zsolt Lengvárszky,** PhD; Louisiana State University Shreveport; Shreveport, LA, USA

**Previous Editor:**

**William F. Finn,** MD - University of North Carolina, USA

